# Differences in Sugar Accumulation and Mobilization between Sequential and Non-Sequential Senescence Wheat Cultivars under Natural and Drought Conditions

**DOI:** 10.1371/journal.pone.0166155

**Published:** 2016-11-04

**Authors:** Huarong Shi, Bin Wang, Piaojuan Yang, Yibo Li, Fang Miao

**Affiliations:** College of Life Sciences, Northwest A&F University, Yangling, Shaanxi, 712100, China; Murdoch University, AUSTRALIA

## Abstract

Wheat leaf non-sequential senescence at the late grain-filling stage involves the early senescence of younger flag leaves compared to that observed in older second leaves. On the other hand, sequential senescence involves leaf senescence that follows an age-related pattern, in which flag leaves are the latest to undergo senescence. The characteristics of sugar metabolism in two sequential senescence cultivars and two non-sequential senescence cultivars under both natural and drought conditions were studied to elucidate the underlying mechanism of drought tolerance in two different senescence modes. The results showed that compared to sequential senescence wheat cultivars, under natural and drought conditions, non-sequential senescence wheat cultivars showed a higher leaf net photosynthetic rate, higher soluble sugar levels in leaves, leaf sheaths, and internodes, higher leaf sucrose synthase (SS) and sucrose phosphate synthase (SPS) activity, and higher grain SS activity, thereby suggesting that non-sequential senescence wheat cultivars had stronger source activity. Spike weight, grain weight per spike, and 100-grain weight of non-sequential senescence cultivars at maturity were significantly higher than those of sequential senescence cultivars under both natural and drought conditions. These findings indicate that the higher rate of accumulation and the higher mobilization of soluble sugar in the leaves, leaf sheaths and internodes of non-sequential senescence cultivars improve grain weight and drought tolerance. At the late grain-filling stage, drought conditions adversely affected leaf chlorophyll content, net photosynthetic rate, soluble sugar and sucrose content, SS and SPS activity, gain SS activity, and weight. This study showed that higher rates of soluble sugar accumulation in the source was one of the reasons of triggering leaf non-sequential senescence, and higher rates of soluble sugar mobilization during leaf non-sequential senescence promoted high and stable wheat yield and drought tolerance.

## Introduction

Some crop cultivars such as wheat and rice undergo sequential leaf senescence. During plant growth, young leaves are successively formed at the top region of the plant, and the lower older leaves gradually undergo senescence [1]. Leaf sequential senescence results from the competition for resources between older and younger leaves [2–5]. During leaf senescence, several leaves are degraded and recycled in the upper plant regions continuously growing parts of the plant, e.g. younger leaves or the forming grains [6–7].

However, some wheat and rice cultivars also display a non-sequential mode of leaf senescence at the grain maturation stage, i.e., younger flag leaves undergo senescence earlier than older second leaves [8–12]. During leaf non-sequential senescence, the chlorophyll content, net photosynthetic rate, transpiration rate, protein content, and catalase and alkaline pyrophosphatase activity of flag leaves are lower than those of the second leaves [8–12]. The rate of phosphate export from flag leaves to grains in non-sequential senescence rice cultivars is generally higher than that involving second leaves. On the other hand, in sequential senescence cultivars, second leaves have higher rates of phosphate export than that in flag leaves [8], thereby demonstrating that phosphorus mobilization from leaves to grains is correlated to leaf senescence. Leaf non-sequential senescence possibly results from a steady increase in abscisic acid analogs in flag leaves [9].

In plants, sugar levels play an important role in regulating plant growth and development. Extensive evidence shows that carbohydrate storage products that accumulate in leaves lower photosynthetic activity and induce leaf senescence [13–22]. However, several other studies support the notion that a reduction in carbohydrate levels triggers senescence [23–29].

Drought is one of the major factors that limit wheat yield, particularly during the grain filling stage. Drought decreases leaf chlorophyll content and net photosynthetic rates, enhances leaf senescence [30], improves stored water-soluble carbohydrates mobilization efficiency in stems, promotes an earlier loss of stem weight [31–34], and shortens the duration of grain filling [35], which in turn result in a reduction in grain number per spike, 1,000-grain weight, straw yield, grain yield, and harvest index [32,36].

In the present study, we compared the chlorophyll content, net photosynthetic rate, soluble sugar content, sucrose content, and the activity of key enzymes during sucrose metabolism in flag leaves and second leaves of sequential and non- sequential senescence cultivars during leaf senescence under natural and drought conditions. The soluble sugar content of leaf sheaths and internodes, spike weight, grain weight per spike, and 100-grain weight were also examined to establish the relationship of sugar accumulation and remobilization with wheat yield, leaf senescence mode, and drought tolerance.

## Materials and Methods

### Plant Materials and Experimental Treatments

Experiments were conducted in the 2013/2014 and 2014/2015 growing seasons at the experimental station of the Northwest A&F University (34°17' N, 108° 05' E), located in the Guanzhong Plain hinterland in Shaanxi Province, which is a part of the winter wheat production area of Huanghuai Plain in China and experiences warm temperate semi-humid climate. Wenmai 19 and Lankaoaizao 8 are non-sequential senescence wheat cultivars that the average grain yields are 7425 and 7125 kg ha^-1^respectivly, and Xinong 88 and NR9405 are sequential senescence wheat cultivars that the average grain yields are 6750 and 6375 kg ha^-1^respectivly under the experimental conditions. Four wheat cultivars were sown in the field on October 6, 2013 and 2014 respectively following a randomized block design with three replicates. Each wheat cultivar was planted in 12 rows that were 23-cm apart and 130-cm long, and plant spacing was 3.33-cm. Before planting, 180 kg N·ha^-1^, 495 kg P_2_O_5_·ha^-1^ were applied. In winter, when the wheat fields were irrigated, 135 kg·ha^-1^ urea was applied. Weeds and insects were adequately controlled during the whole wheat growing period. The field was exposed to two ecological conditions, namely, drought conditions that prevented rain from entering the field by constructing a white plastic shed from wheat heading stage to maturity, and natural conditions, which allowed rain entering field in the same stage. In the natural conditions, the average soil water content of 0–20, 20–40, 40–60 cm soil depth was 17.46%, 13.13%, 12.35%, respectively, and in the drought conditions, was 7.51%, 10.12%, 12.23%, respectively.

The anthesis date of Xinong 88, NR9405, Wenmai 19 and Lankaoaizao 8 was April 26, April 27, April 28, and April 28, 2015, respectively. At the anthesis stage, the stalks of the same anthesis day were tagged, and served as sources of test samples. At 15, 20, 25, 30 days after anthesis, from 9:00 AM to 11:00 AM, the net photosynthetic rate of nine flag leaves and nine second leaves was measured for per cultivar and per treatment. Simultaneously, the leaves, leaf sheaths, internodes, and grains of fifteen stalks for per cultivar and per treatment were collected and oven-dried to determine soluble sugar and sucrose content, whereas the leaves and grains of fifteen stalks that were used for the determination of chlorophyll and enzyme activity were weighed and then stored in a -80°C refrigerator after flash freezing in liquid nitrogen. At the anthesis stage, 20 days after anthesis, and maturity, sixty spikes for per cultivar and per treatment were collected and oven-dried for estimation of spike weight, grain weight per spike, and 100-grain weight. The mature date of Xinong 88, NR9405, Wenmai 19 and Lankaoaizao 8 was May 28, May 30, June 2, and June 5, 2015, respectively.

### Chlorophyll Content

Total chlorophyll was extracted from frozen leaf samples (~0.2 g) with three replications using 80% acetone at room temperature until the tissue was completely bleached. The extract was centrifuged at 5,000*g* for 10 min, and the absorbance of the supernatant was measured at wavelengths of 645 nm and 663 nm using a spectrophotometer. Total chlorophyll content was calculated by using the following formula: Total chlorophyll content = 20.21A_645_ + 8.02A_663_, as described by Arnon [37] and Chen [38]. The chlorophyll content (mg·g^-1^ DW) was then calculated.

### Net Photosynthetic Rate

Net photosynthetic rates of leaves were measured in the field from 9:00 AM to 11:00 AM with a portable photosynthesis system (Li-6400; LI-COR Inc., Lincoln, NE, USA). The flag leaf or the second leaf was placed in the chamber at a photon flux density of 1,000 μmol·m^-2^·s^-1^; the flow rate through the chamber was 500 μmol·s^-1^, and leaf temperature was approximately 28°C. The ambient CO_2_ concentration was approximately 400 μmol CO_2_·mol^-1^ air, and vapor pressure deficit was maintained at approximately 2.0 kPa [38]. Nine leaves from each cultivar were measured.

### Soluble Sugar and Sucrose Content

Dried samples were ground to a fine powder for soluble sugar and sucrose analysis. The sample powder (~0.2g) with three replications was extracted using 6 mL of 80% (v/v) ethanol for 30 min in a water bath at 80°C, then the supernatant was collected after centrifugation at 5,000*g* for 10 min. This extraction procedure was repeated three times. The three supernatants were pooled and then diluted with 80% ethanol to 25 mL for the measurement of soluble sugar and sucrose content. Soluble sugar content was determined by using the anthrone reagent method and calculated based on the absorbance at a wavelength of 625 nm and a standard curve [39]. Sucrose content was measured by using the resorcinol method and estimated on the basis of the absorbance at a wavelength of 480 nm and a standard curve [40]. The mobilized soluble sugar content was calculated by the difference between the largest sugar content and the sugar content at 30 days after anthesis.

### Measurement of Sucrose Synthase (SS) (Synthetic Direction) and Sucrose Phosphate Synthase (SPS) Activities

Each frozen leaf sample (~0.2 g) with three replications was placed in a porcelain mortar and homogenized to a fine powder in liquid nitrogen. Approximately 2 mL of HEPES/NaOH (pH 7.5) buffer was then added for enzyme extraction in an ice bath, and then centrifuged at 11,000*g* at 4°C for 10 min. The supernatant was used for the measurement of both SS (synthetic direction) and SPS activities [41–42].

SS (synthetic direction) activity: The reaction mixture containing 50 μL of the enzyme solution, 50 μL of 50 mM HEPES-NaOH (pH 7.5), 20 μL of 50 mM MgCL_2_, 20 μL of 100 mM UDPG, and 20 μL of 100 mM fructose was incubated for 30 min at 30°C. After incubation, 200 μL of 2 M NaOH was added and the mixture was heated in boiling water for 10 min. After allowing the mixture to cool down, 1.5 mL of 30% HCI and 0.5 mL of 0.1% resorcinol were added, which was followed by incubation for 10 min at 80°C. After cooling, the absorbance of the reaction solution was measured at a wavelength of 480 nm. SS activity was calculated according to the standard carve and expressed in mg sucrose synthesized at g^-1^ FW·h^-1^. For the blank, UDPG and fructose were replaced by distilled water and the same measurement procedure for SS activity was performed [41–42].

SPS activity: The reaction mixture and procedures were similar to that used in estimating SS (synthesis direction) activity except that fructose was replaced with fructose-6-phosphate. SPS activity was expressed in mg sucrose synthesized at g^-1^ FW·h^-1^. UDPG and fructose-6-phosphate were replaced by distilled water for blank measurement [43].

### The Activity of SS (Cleavage Direction)

SS (cleavage direction) activity was measured according to Douglas [41]. Each frozen grain sample (~ 0.5 g) with three replications was placed in a porcelain mortar and homogenized to a fine powder in liquid nitrogen. Approximately 5 mL of ice-cold extraction buffer containing 50 mM HEPES-NaOH (pH 7.5), 5 mM MgCl_2_, and 1 mM DTT was then added, and the mixture was centrifuged at 11,000*g* for 15 min at 4°C. The supernatant was then collected and used for the measurement of SS activity in the cleavage direction. The enzyme reaction mixture containing 100 μL of enzyme extract and 100 μL of a mixture containing 100 mM HEPES-NaOH (pH 6.0), 100 mM sucrose, and 10 mM UDP was incubated for 30 min in a 30°C water bath, and the reaction was terminated by boiling for 5 min. Fructose production was measured at a wavelength of 510 nm using a dinitrosalicylic acid solution. An assay mixture not containing UDP was used as control. SS activity in the cleavage direction was expressed in mg fructose produced at g^-1^ FW·h^-1^.

### Spike Weight, Grain Weight per Spike, 100-grain Weight

At anthesis, 20 days after anthesis, and maturity (40 days after anthesis), 20 spikes from each plot were collected and dried in an 85°C oven for 48 h for the determination of spike weight, grain weight per spike, and 100-grain weight. Spike weight gain and grains per spike weight gain from anthesis to 20 days after anthesis and from 20 days after anthesis to maturity were calculated based on the difference in weight.

### Statistical Analysis

Data were analyzed by analysis of variance (ANOVA) in SPSS statistics software (Version 19.0 for Windows, SPSS, Chicago, USA). Differences between the mean values were compared using LSD and Duncan, and a *P* value ≤ 0.05 was considered significant.

## Results

### Sequential and Non-sequential Senescence of Wheat Leaves under Natural and Drought Conditions

**Fig 1:**
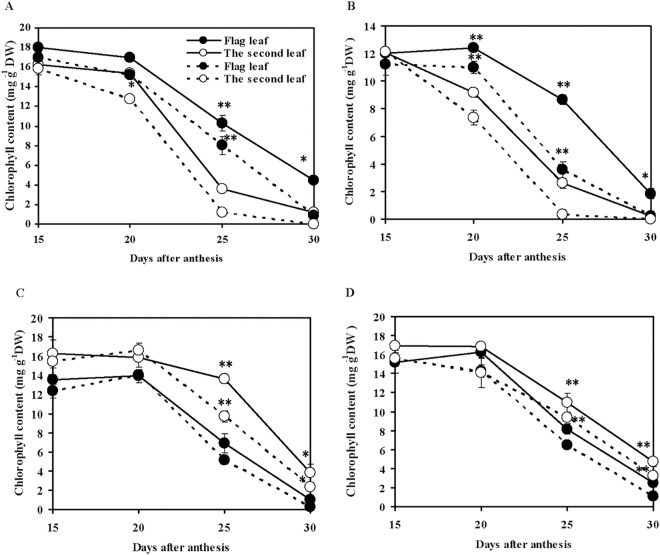
Changes in chlorophyll content of flag leaf and the second leaf of sequential and non-sequential senescence wheat cultivars under natural and drought conditions. (A) Xinong 88, sequential senescence wheat cultivar. (B) NR9405, sequential senescence wheat cultivar. (C) Wenmai 19, non-sequential senescence wheat cultivar. (D) Lankaoaizao 8, non-sequential senescence wheat cultivar. Solid and dash lines represent natural and drought conditions. Each bar represents the mean ± SD of three replications. Asterisks indicate statistically significant difference between flag leaf and the second leaf in the same conditions (*P ≤ 0.05, ** *P* ≤ 0.01).

Fig 1 shows the changes in chlorophyll content of the flag and second leaves of the four wheat cultivars from 15 to 30 days after anthesis under natural and drought conditions. In Xinong 88 and NR9405 (Fig 1A and 1B), the flag leaf showed higher chlorophyll contents than the second leaf during leaf senescence, indicating that the second leaf senesced earlier than the flag leaf, which we designated as wheat leaf sequential senescence. In Wenmai 19 and Lankaoaizao 8 (Fig 1C and 1D), the second leaf showed higher chlorophyll content than the flag leaf during leaf senescence, named as wheat leaf non-sequential senescence. At 25 days after anthesis, a highly significant difference in leaf color between sequential and non-sequential senescence was observed, i.e., the flag leaf was green and the second leaf was yellow in sequential senescence cultivars, but in non-sequential senescence cultivars, the second leaf was green and the flag leaf was yellow. Compared to natural conditions, drought conditions significantly increased the rate of the loss of leaf chlorophyll, indicating that leaf senescence was accelerated.

### Changes in Net Photosynthetic Rate of Flag Leaf and the Second Leaf under Natural and Drought Conditions

**Fig 2:**
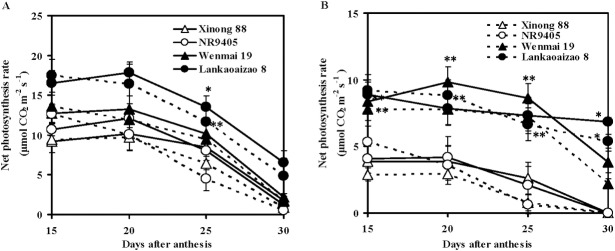
Changes in net photosynthetic rate of flag leaf and the second leaf of sequential and non-sequential senescence wheat cultivars under natural and drought conditions. (A) Flag leaf, (B) The second leaf. Solid and dash lines represent natural and drought conditions. Each bar represents the mean ± SD of nine replications. Asterisks indicate statistically significant difference between sequential and non-sequential senescence wheat cultivars in the same conditions (*P ≤ 0.05, ** *P* ≤ 0.01).

Under natural conditions, the net photosynthetic rate of flag leaf of Lankaoaizao 8 (non-sequential senescence cultivar) was significantly higher than those of Xinong 88 and NR9405 (sequential senescence cultivars), and that of the non-sequential senescence cultivar Wenmai 19 was slightly higher (Fig 2A). Under drought conditions, the net photosynthetic rate of the flag leaves of both Xinong 88 and NR9405 was rapidly and significantly reduced compared to those of Lankaoaizao 8 and Wenmai 19 from 15 to 30 days after anthesis (Fig 2A). The net photosynthetic rates of the second leaf of Lankaoaizao 8 and Wenmai 19 were significantly higher than those of Xinong 88 and NR9405 under both natural and drought conditions (Fig 2B). These findings indicated that non-sequential senescence wheat cultivars had higher leaf net photosynthetic rates during grain filling stages, which is then conducive to an increase in grain weight.

### Changes in Soluble Sugar Content of Leaves, Leaf Sheaths, and Internodes under Natural and Drought Conditions

**Fig 3:**
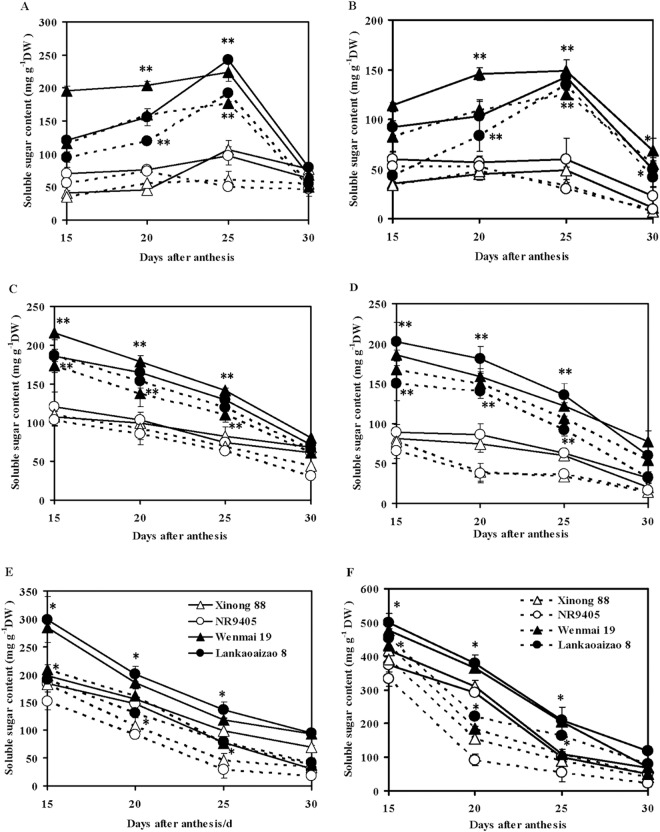
Changes in soluble sugar content of the leaf, leaf sheath, and internode of sequential and non-sequential senescence wheat cultivars under natural and drought conditions. (A) Flag leaf, (B) The second leaf, (C) Flag leaf sheath, (D) The second leaf sheath, (E) Peduncle, (F) Penultimate internode. Solid and dash lines represent natural and drought conditions. Each bar represents the mean ± SD of three replications. Asterisks indicate statistically significant difference between sequential and non-sequential senescence wheat cultivars in the same conditions (*P ≤ 0.05, ** *P* ≤ 0.01).

A significant difference in leaf soluble sugar content between sequential and non-sequential senescence cultivars was observed. The soluble sugar content of flag leaves and the second leaves of non-sequential senescence cultivars was significantly higher than those of sequential senescence cultivars in both natural and drought conditions (Fig 3A and 3B). In natural conditions, the soluble sugar content of the flag leaves and the second leaves gradually increased from 15 to 25 days after anthesis, and that from 25 to 30 days rapidly decreased. The mobilized soluble sugar content of the flag leaves and the second leaves of non-sequential senescence cultivars was significantly higher than those of sequential senescence cultivars. The mobilized soluble sugar content of the flag leaves of Wenmai 19, Lankaoaizao 8, Xinong 88, and NR9405 were 156.74 mg/g, 163.46 mg/g, 29.54 mg/g, and 34.52 mg/g, respectively; in the second leaves this was 80.72 mg/g, 92.86 mg/g, 37.98 mg/g, and 37.49 mg/g, respectively. In drought conditions, the accumulated soluble sugar content in flag leaves and the second leaves significantly decreased. The time to reach the peak soluble sugar content in the flag leaves and the second leaves of non-sequential senescence cultivars was the same as that observed in natural conditions at 25 days after anthesis, but that of sequential senescence cultivars was earlier by 5 days or more. These findings suggested that the higher accumulation and mobilization of soluble sugar in leaves of non-sequential senescence cultivars during the grain-filling stage might help to improve grain weight and drought tolerance.

The soluble sugar content of leaf sheaths and internodes decreased from 15 to 30 days after anthesis, which differed from that observed in the leaves (Fig 3C–3F). The soluble sugar content of leaf sheaths and internodes of non-sequential senescence cultivars was significantly higher than that of sequential senescence cultivars. In natural conditions, from 15 to 30 days after anthesis, the mobilized soluble sugar content in flag sheath, the second leaf sheath, peduncle, and penultimate internode of non-sequential senescence cultivars Wenmai 19 and Lankaoaizao 8 were 135.65 and 114.55 mg/g, 108.32 and 142.3 mg/g, 191.82 and 203.54 mg/g, 405.08 and 382.14 mg/g, respectively, and that of sequential senescence cultivars Xinong 88 and NR9405 were 39.32 and 59.49 mg/g, 61.55 and 56.46 mg/g, 127.8 and 152.34 mg/g, 351.09 and 324.88 mg/g, respectively. Compared to natural conditions, the high degradation level of soluble sugar in the leaf sheaths and internodes under drought conditions indicated that drought conditions accelerated soluble sugar transport from the leaf sheaths and internodes to the grains. These findings indicated that the higher accumulation and higher reduction of soluble sugar in leaf sheaths and internodes of non-sequential senescence cultivars might help to improve grain weight and drought tolerance.

### Changes in Leaf Sucrose Content, SS and SPS Activities under Natural and Drought Conditions

**Fig 4:**
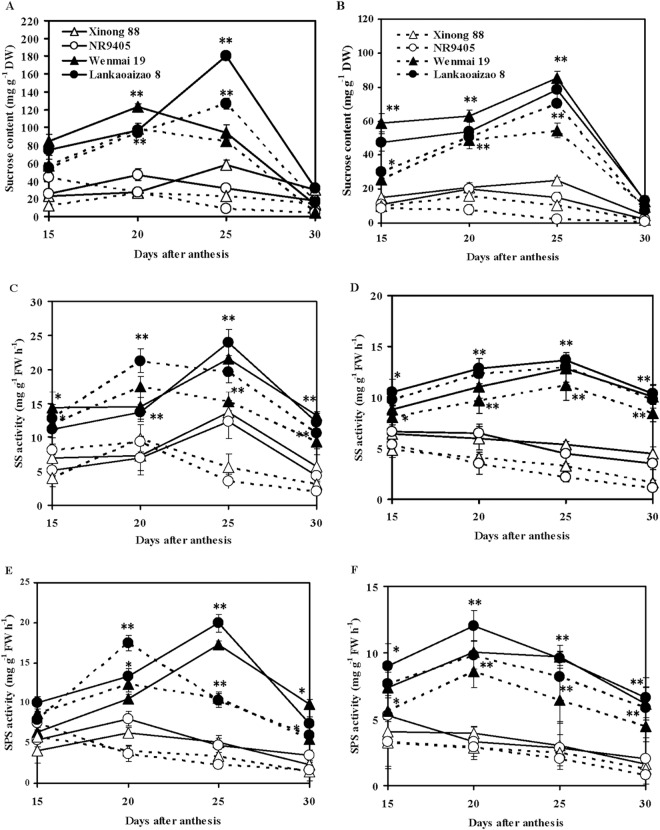
Changes in leaf sucrose content, SS and SPS activities of sequential and non-sequential senescence wheat cultivars under natural and drought conditions. (A), (C) and (E) Flag leaf. (B), (D) and (F) The second leaf. Solid and dash lines represent natural and drought conditions. Each bar represents the mean ± SD of three replications. Asterisks indicate statistically significant difference between sequential and non-sequential senescence wheat cultivars in the same conditions (*P ≤ 0.05, ** *P* ≤ 0.01).

Under natural conditions, the sucrose content of flag leaves and second leaves initially increased, and then gradually decreased. The time to reach the peak sucrose content was 20 days or 25 days after anthesis (Fig 4A and 4B). The sucrose content of flag leaves and second leaves of non-sequential senescence cultivars was significantly higher than that of sequential senescence cultivars. The peak sucrose content of flag leaves of non-sequential senescence cultivars Wenmai 19 and Lankaoaizao 8 was 122.79 and 179.97 mg/g, that in the second leaves were 85.37 and 78.34 mg/g, respectively. Peak sucrose content of flag leaves of sequential senescence cultivars Xinong 88 and NR9405 was 57.77 and 47.03 mg/g, those of second leaves was 24.95 and 19.97 mg/g, respectively. Under drought conditions, Peak sucrose content of flag leaves and second leaves markedly decreased, the time for flag leaves and second leaves of non-sequential senescence cultivars to reach the highest sucrose content was the same as that observed in natural conditions, whereas for sequential senescence cultivars, this was earlier by 5 days or more, which indicated that the non-sequential senescence cultivars had higher photosynthesis capacity and sucrose synthesis capacity than the sequential senescence cultivars under drought conditions.

The SS activities (in the synthesis direction) of flag leaves and second leaves of non-sequential senescence cultivars were generally higher than those of sequential senescence cultivars from 15 to 30 days after anthesis regardless of being exposed to natural or drought conditions (Fig 4C and 4D). Under natural conditions, SS activity of flag leaves initially increased and then sharply decreased from 25 days after anthesis. On the other hand, the second leaves exhibited a differ enzyme activity pattern between sequential and non-sequential senescence cultivars. The non-sequential senescence cultivars showed an initial slow increase in enzyme activity, which was then followed by a gradual decline from 25 days after anthesis, whereas the sequential senescence cultivars presented a slow decrease in from 15 to 30 days after anthesis, which indicated that the second leaf of sequential senescence cultivars enter senescence earlier. Compared to natural conditions, drought conditions enhanced SS activities of flag leaves from 15 to 20 days after anthesis and decreased these from 20 to 30 days after anthesis, which showed that drought enhanced photosynthate transport from flag leaves to grains. Drought conditions significantly reduced SS activities of the second leaves from 15 to 30 days after anthesis. SS activity was significantly correlated with sucrose content of leaves. The correlation coefficient was 0.854**, suggesting that the increase in sucrose content in leaves was associated with enhanced SS activity.

The SPS activities of flag leaves and second leaves of non-sequential senescence cultivars were also higher than those of sequential senescence cultivars from 15 to 30 days after anthesis under both natural and drought conditions (Fig 4E and 4F). Under natural conditions, the SPS activity of flag leaves initially increased and then decreased from 20 days or 25 days after anthesis. The SPS activity of the second leaves of non-sequential senescence cultivars showed the same changes as that observed in flag leaves, but those of sequential senescence cultivars slowly decreased from 15 to 30 days after anthesis. Compared to natural conditions, drought conditions enhanced the SPS activity of flag leaves at the early grain-filling stage, which was followed by a decrease at the late grain-filling stage. Drought conditions greatly reduced SPS activity of second leaves. SPS activity was also significantly correlated with the sucrose content of leaves. The correlation coefficient was 0.846**, suggesting that the increase in sucrose content of leaves was associated with enhanced SPS activities.

### Spike Weight, Grain Weight per Spike, 100-grain Weight, and Grain SS Activities under Natural and Drought Conditions

**Fig 5:**
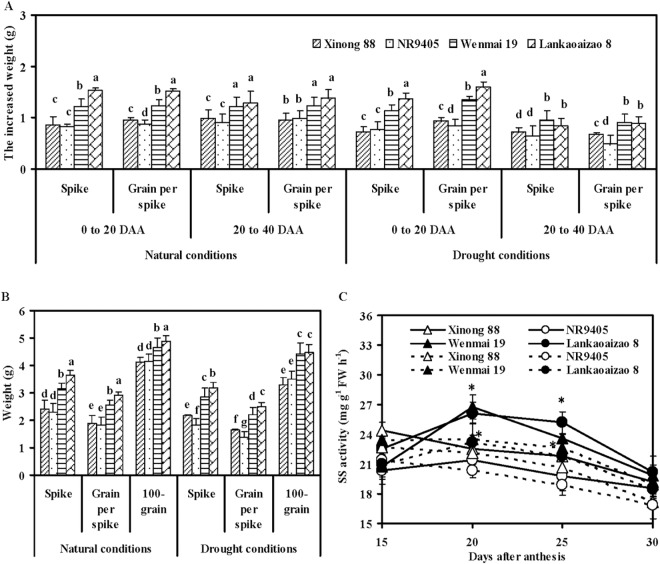
Spike weight, grain weight per spike, 100-grain weight, and grain SS activities (cleavage direction) of sequential and non-sequential senescence wheat cultivars under natural and drought conditions. (A) The increased weight in spike and grain per spike from 0 to 20 days after anthesis (DAA) and 20 to 40 DAA (maturity), (B) Spike weight, grain weight per spike, and 100-grain weight at maturity, (C) grain SS activities (cleavage direction). Solid and dash lines represent natural and drought conditions. Each bar represents the mean ± SD of three replicates. Different letters indicate significant difference (*P* ≤ 0.05) between two treatments and among four cultivars. Asterisks indicate significant difference between sequential and non-sequential senescence wheat cultivars in the same conditions (*P ≤ 0.05, ** *P* ≤ 0.01).

At 20 days after anthesis, the flag leaves and second leaves showed signs of rapid senescence in both sequential and non-sequential senescence cultivars. Whether from 0 to 20 days after anthesis or 20 days to maturity (40 days after anthesis), the increased weight in spike and grain per spike of non-sequential senescence cultivars was significantly higher than that of sequential senescence cultivars (Fig 5A). In natural conditions, compared to sequential senescence cultivars, the mean increased weight in spike and grain per spike of non-sequential senescence cultivars was higher by 64.29% and 50.27%, respectively, from 0 to 20 days after anthesis, and higher by 32.73% and 34.39%, respectively, from 20 days to 40 days. Under drought conditions, these were higher by 67.79% and 14.84%, respectively, from 0 to 20 days after anthesis, and higher by 30.66% and 52.99%, respectively, from 20 days to 40 days. These findings indicated that leaf non-sequential senescence in wheat was conducive to an increase in spike and grain per spike weight at the early and late grain-filling stages under both natural and drought conditions. Drought conditions had no significant effect on the increased weight in spike and grain per spike from 0 to 20 days after anthesis, but significantly decreased the increased weight in spike and grain per spike from 20 to 40 days after anthesis.

Spike weight, grain weight per spike, and 100-grain weight of non-sequential senescence cultivars at maturity were significantly higher than those of sequential senescence cultivars under both natural and drought conditions (Fig 5B). Drought conditions significantly decreased spike weight, grain weight per spike, and 100-grain weight by an average of 11.07%, 14.39%, and 6.51% in non-sequential senescence cultivars, respectively, and by an average of 15.44%, 19.11%, and 17.72% in sequential senescence cultivars, thereby suggesting that non-sequential senescence cultivars had higher grain yield and stronger drought tolerance.

From 20 to 30 days after anthesis when flag leaves and second leaves undergo rapid senescence, the grains of non-sequential senescence cultivars showed higher SS activity (cleavage direction) compared to that of sequential senescence cultivars under both natural and drought conditions (Fig 5C), indicating that non-sequential senescence cultivars had stronger sink activity for sucrose unloading at the late grain filling stage. Drought-accelerated grain senescence resulted in a reduction in grain SS activity.

## Discussion

In the late grain filing stage, wheat cultivars Wenmai 19 and Lankaoaizao 8 showed a non-sequential senescence mode, i.e., younger flag leaves senesced earlier than the older second leaves. However, wheat cultivars Xinong 88 and NR9405 showed a leaf sequential senescence mode that was age-related, similar to that reported in rice [8–10]. During leaf non-sequential senesce, chlorophyll, protein, and RNA contents, as well as catalase activity of flag leaves were lower compared to those of second leaves [8–10]. On the other hand, the rate of ^32^P-phosphate export was higher, thereby suggesting that flag leaves undergo accelerated senescence, which might be due to the enhancement in photosynthate transport to grains [8].

Sugar status modulates and coordinates internal regulators and environmental cues that govern plant growth and development. Several lines of evidence suggest that low sugar levels induce leaf senescence. Photosynthetic rates decrease prior to leaf yellowing, thereby indicating that developmental leaf senescence is induced by low sugar levels [16]. Low sugar levels increase ethylene production or sensitivity, and ethylene acts as an accelerator of leaf senescence [26]. Monocarpic senescence apparently occurs as a consequence of reproduction, and leaf sequential senescence seems to result from competition for resources between older and younger leaves. Leaves of various monocarpic species live longer when flowering and pollination are inhibited [2]. Senescence in soybean or spinach plants is delayed upon the removal of their flowers or fruits [3]. In the present study, sequential senescence wheat cultivars Xinong 88 and NR9405 leaves showed lower soluble sugar content during the grain filling stage, when their leaf net photosynthetic rates decreased from 20 days after anthesis to maturity, competition for sugar between leaves and grains was more intense, and leaf senescence was induced based on age. However, several lines of evidence support the notion that leaf senescence is caused by elevated sugar levels in the cell rather than sugar starvation [13–22]. When carbohydrate storage products accumulate in the leaves of tobacco, *Arabidopsis*, and *Medicago*, a decline in the rate of photosynthesis and premature yellowing is observed [17, 44]. Under long days, moderate light leads to early accumulation of hexoses compared to low light conditions, and the start of hexose accumulation coincides with a decline in chlorophyll content, thereby indicating that increased light intensity triggers senescence due to the earlier accumulation of hexoses [45]. In our experiments, the leaves of non-sequential senescence wheat cultivars Wenmai 19 and Lankaoaizao 8 showed higher net photosynthetic and soluble sugar accumulation rates during the grain filling stage, which in turn triggered flag leaves to undergo senescence earlier than that observed in second leaves, as well as accelerated the transport of soluble sugar from the flag leaves to grains. In conclusion, at the grain filling stage when the level of soluble sugar in wheat leaves is low, leaves undergo sequential senescence. On the other hand, when the level soluble sugar in wheat leaves is high, leaves undergo non-sequential senescence to accelerate the transport of soluble sugars from flag leaves to grains.

In cereals such as wheat and rice, grain filling utilizes carbon that is derived from photosynthetic assimilation and the remobilization of prestored carbohydrates from mature leaves, leaf sheaths, and culms. Nonstructural carbohydrates (soluble sugars and starch) in leaf sheaths and culms play an important role in compensating for the low carbon supply via photosynthetic accumulation after heading [46]. Approximately 30% of the total yield depends on the carbohydrates that had accumulated in the stem prior to heading [47]. When photosynthesis decreases due to various adverse conditions after heading, the carbohydrates that accumulated before heading is utilized in grain filling. The transfer ratio of carbohydrates that are pre-stored in the stems and its contribution to grain filling from the stages of heading to maturity in dry-cultivated rice is significantly higher than that in moist-cultivated rice [48]. Increasing the capacity of leaves to store a higher amount of carbohydrates is one of the main targets in improving crop yield [49]. Carbohydrate accumulation is particularly important for grain filling in high-yielding cultivars that bear a large number of spikelets [50–52]. The results in the present study showed that leaves, leaf sheaths, and internodes of non-sequential senescence wheat cultivars had significantly higher soluble sugar content compared to that of sequential senescence wheat cultivars under both natural and drought conditions, which was beneficial to grain filling. This feature possibly also compensates for the lack of carbohydrate supply due to a decrease in the rate of leaf photosynthesis during drought. Therefore, leaf non-sequential senescence at the late grain-filling stage is a high and stable yield character that could be used in wheat breeding programs.

Sucrose is the main source of carbon and energy in the sink tissues of plants. It is synthesized in the cytoplasm of leaves (source tissues) from where it is loaded into the phloem and transported to sink tissues. After sucrose transport and accumulation in storage tissues, various enzymes engage in various reactions related to sugar metabolism. SS uses uridine diphosphate (UDP)-glucose and fructose to synthesize sucrose in plant leaves [53]. The same enzyme in sink tissues acts in the reverse direction to convert sucrose into UDP-glucose and fructose. SPS synthesizes sucrose phosphate, which is then converted to sucrose by sucrose phosphate phosphatase in the source tissues and then loaded into the phloem [54]. Higher leaf SS and SPS activity reflects the ability of leaves to convert photosynthates into sucrose, which in turn represents source strength [54]. The ability of sink tissues to accept and convert photoassimilates is known as sink strength. The degradation of sucrose in sink tissues is mainly catalyzed by SS; therefore, SS cleavage direction activity serves as an indicator of sink strength [55–57]. In the present study, sucrose content and SS and SPS activities of flag leaves and second leaves of non-sequential senescence wheat cultivars were significantly higher than those of sequential senescence wheat cultivars under both natural and drought conditions, thereby suggesting that non-sequential senescence wheat cultivars possessed stronger source activity that ensures that sufficient sucrose is loaded into the phloem during the grain-filling stage (Fig 4). Grain SS activities of non-sequential senescence wheat cultivars were also significantly higher than those of sequential senescence wheat cultivars, showing that non-sequential senescence wheat cultivars had stronger sink activity for unloading sucrose (Fig 5C). The higher spike weight, grain weight per spike, and 100-grain weight of non-sequential senescence cultivars under both natural and drought conditions may be attributable to higher source and sink activity. From 20 to 30 days after anthesis, the chlorophyll content of flag leaves and second leaves sharply decreased, indicating that wheat plants underwent rapid senescence (Fig 1). However, during this stage, flag leaves, second leaves, and grains of non-sequential senescence cultivars continued to show higher SPS and SS activity, which are responsible for the observed higher spike weight gain at the late grain-filling stage (Figs 4 and 5).

## Supporting Information

S1 Fig Changes in chlorophyll content of the flag leaf and the second leaf of sequential and non-sequential senescence wheat under natural and drought conditions. (XLS)

S2 Fig Changes in net photosynthetic rate of flag leaves and the second leaves under natural and drought conditions. (XLS)

S3 Fig Changes in soluble sugar content of the leaf, leaf sheath, and internode under natural and drought conditions. (ZIP)

S4 Fig Changes in leaf sucrose content and SS and SPS activities under natural and drought conditions. (ZIP)

S5 Fig Spike weight, grain weight per spike, 100-grain weight, and grain SS activities (cleavage direction) under natural and drought conditions. (ZIP)
